# Does combination of estradiol and sesame oil improve the oocyte quality, embryo development and expressions of *Zp3, E-cad,* and *Ctnnb1* genes in mice? An experimental study

**DOI:** 10.18502/ijrm.v19i8.9618

**Published:** 2021-09-09

**Authors:** Masoomeh Mohammadzadeh, Fatemeh Anbari, Shiva Aghaei, Ehsan Farashahi Yazd, Zhima Akhavan Sales, Mahya Rajabi, Mohammad Ali Khalili

**Affiliations:** ^1^Research and Clinical Center for Infertility, Yazd Reproductive Sciences Institute, Shahid Sadoughi University of Medical Sciences, Yazd, Iran.; ^2^Department of Reproductive Biology, Faculty of Medical Sciences, Shahid Sadoughi University of Medical Sciences, Yazd, Iran.; ^3^Stem Cell Biology Research Center, Yazd Reproductive Sciences Institute, Shahid Sadoughi University of Medical Sciences, Yazd, Iran.; ^4^Department of Immunology, Faculty of Medicine, International Campus, Shahid Sadoughi University of Medical Sciences, Yazd, Iran.; ^5^Abortion Research Center, Yazd Reproductive Sciences Institute, Shahid Sadoughi University of Medical Sciences, Yazd, Iran.

**Keywords:** Estradiol, Sesame oil, Gene expression, Oocyte, Mouse.

## Abstract

**Background:**

Aging may reduce oocyte maturation, embryo quality, and fertility potential.

**Objective:**

To compare the effect of estradiol (E2) and sesame oil on oocyte and embryo quality between young and old mice.

**Materials and Methods:**

Sixty old and young female mice were divided in to two groups (30 mice/group, grouped by age). Each group was divided into three subgroups of mice treated with sesame oil, E2 + sesame oil, and normal saline as control group. After ovulation induction, some oocytes were considered for in vitro fertilization and the rest were assessed for morphological status. After obtaining the two-cell embryos, the embryos were collected to determine the expression of zona pellucida (ZP) glycoprotein 3, *E-cadherin*, and β-*catenin* genes and some of them followed until the blastocysts stage to evaluate the viability.

**Results:**

The findings showed that the mean ZP and perivitelline space thickness increased in the old mice that received the E2 + sesame oil treatment. The number of 2-cell embryos, blastocysts, and live cells were significantly higher in the old group treated with sesame oil respectively (p = 0.018, 0.002, and < 0.0001, respectively). The normal ZP shape and refractile body numbers increased in the old mice that were treated with sesame oil, respectively. The *E-cadherin* gene was downregulated in the treatment groups compared to the controls.

**Conclusion:**

Sesame oil showed a better response in the old mice, because aging is associated with an increased rate of reactive oxygen species, causing deficiencies in both oocyte and embryo qualities.

## 1. Introduction

Cytoplasmic morphology and maturation of oocytes depend on both intrinsic (e.g., age, genetic) and extrinsic (e.g., stimulation protocol) factors. These protocols may influence extraooplasmatic features, such as the perivitelline space (PVS) and zona pellucida (ZP). The ZP and PVS integrity can play a vital role in the relationship between oocyte and granulosa cells as well as cytoplasmic maturity (1, 2). So, both ZP and PVS structures' probability have important roles in transmitting metabolic signals into the oocyte (3). Aging as an intrinsic factor reduces the levels of both estrogen and antioxidants in the ovary (4). It has also been proven that aging reduces the rates ‎of fertilization and embryo development (5, 6).

In addition to estrogen, sesame oil contains large amounts of polyunsaturated fatty acids with antioxidant activity and vitamin E, which is proven to prevent DNA oxidative damage (7). Sesame oil is an important source of phytoestrogens with estrogenic properties (8). Furthermore, it appears that the success of in vitro fertilization (IVF) depends on the structural and functional quality of oocytes and the ZP. The ZP glycoprotein 3 (ZP3) is the primary sperm receptor: it plays a key role in sperm binding to the oocyte and inducing the acrosome reaction (9). Lack of *ZP3 *due to the absence of zona matrix prevents fertilization and embryo formation (10). Studies have shown that steroid hormones have positive effects on oocyte growth, developing embryos and expression of adhesion molecules, such as E-cadherin (*E-cad)*. Cadherins are genes of calcium-dependent membrane glycoproteins that mediate cell adhesion (11). *E-cad* plays a mediating role in blastomere compaction at the 8-cell stage and catenins mediate cadherin activity (12). However, deletion of the catenin or adherins-binding site damages the cellular reorganization and adhesion function. Therefore, it can be concluded that steroid hormones not only affect the morphology and quality of oocytes and embryos but also play a role in the gene expression of cadherin and catenin family members (13). However, to the best of our knowledge, no previous study has evaluated the positive effects of the simultaneous use estradiol E2 and sesame oil on ovarian cycles in old mice. Hence, we decided to inject E2 + sesame oil and sesame oil alone at a time when the level of maternal estrogen is at its lowest to assess oocyte quality and embryo development, as well as the expression of *Zp3*, *E-cad*, and β-catenin (*Ctnnb1*) ‎genes in both old and young mice.

## 2. Materials and Methods

### Subjects

Initially, 90 NMRI female mice were included in this prospective study. Of these, 30 old mice were excluded due to lack of response to ovarian stimulation and defective uterine cycle. Finaly, sixty old and young mice (30 mice/group, grouped by age) were examined. Mice aged six-eight and 35-40 wk were considered as young and old mice, respectively (14). The mice lived in standard conditions of 22 ± 2°C and a 12-hr light/dark cycle. Both young and old groups were categorized into three subgroups: controls receiving normal saline, experimental mice receiving 1-µl E2 + sesame oil (10 µl/kg/day) and sesame oil alone (10 µl/kg/day) (15). In each group, the treatment fluids were injected by the intraperitoneal (IP) injection method (16).

### Determination of estrus cycle

The vaginal smears were evaluated under an optic microscope. The estrus phase (proestrus [A], estrus [B], metestrus [C] and diestrus [D]) of each mouse was defined according to the previous report (17). So, 50 µl of sterile normal saline was injected into the vagina, and following aspiration, the smears were prepared and stained with Coomassie Blue staining. The duration of each stage was evaluated from the vaginal smears. The E2 was administrated at the time of the diestrus cycle, when the E2 level was low (18). So, all samples received their respective treatment in diestrus. The following day (in estrus), after superovulation, 10 IU of pregnant mare serum gonadotropin (PMSG, VWR Scientific Inc, USA) was injected IP, and 48 hr later, 10 IU of human chorionic gonadotropin (hCG, OvitrelleⓇ, Merck Serono, Germany) was injected IP.

### In vitro fertilization

Mice were euthanized 16 hr later by cervical dislocation. Oviducts were removed and the cumulus-oocyte complexes (COCs) were retrieved from the oviducts and transferred to a G-IVF medium (Vitrolife Co., Sweden). Half of the oocytes denuded by hyaluronidase (80 IU/ml, LifeGlobal, Belgium) were evaluated under an inverted microscope to assess the morphological parameters according to granularity (large or small granules; homogenous or clustering distribution of granules), size of PVS and ZP, ZP abnormalities (dark, duplication of the inner layer, thick ZP), and distribution of organelles (vacuoles, endoplasmic reticulum, and refractile bodies [RFs]). The thickness of the PVS (µm) and ZP (µm) was also evaluated from three different areas of oocytes by the morphometric software and inverted microscope (Nikon, Japan) and the average sizes were recorded.

The other half of the COCs were retrieved from the oviducts and transferred to the G-IVF medium. A male mouse was killed and the cauda epididymis was removed. The sample was cut using a pair of syringes to release the spermatozoa and was left in an incubator at 37°C and 5% CO${}_{2}$ for 60 min. Then, 10 µl of sperm sample was added to the drop of COCs and the fertilization dish was placed in an incubator for four-five hr. Finally, the COCs were denuded and the fertilized oocytes were transferred to a G1 medium (Vitrolife Co., Sweden). Next, 48 hr after the fertilization, 2-cell embryos were vitrified for future analysis.

### Vitrification of embryos 

“Two-cell embryos were incubated in an equilibration solution comprising with Ham's F-10 media with 20% human serum albumin (HAS) that supplemented with 7.5% ethylene glycol (Sigma-Aldrich, Germany) and 7.5% dimethyl sulphoxide (Sigma-Aldrich, Germany) for 5-15 min at room temperature (RT) condition. After then, they were placed into the vitrification solution containing Ham's F-10 medium supplemented with 15% ethylene glycol, 15% dimethyl sulphoxide and 0.5 M sucrose (Merck, Darmstadt, Germany) for 50-60 sec at RT. Eventually, the embryos placed on the tip of the Cryotop (Kitazato, Japan) for storage in liquid nitrogen. Warming of embryos was performed by placing the Cryotop in TS solution (1 M sucrose) for 50-60 sec at RT and then into a dilution solution (0.5 M sucrose) for 3 min. The warmed embryos were placed into a washing solution (Ham's F-10 and 20% HSA) four times" (19). They were divided into two groups. Half of the embryos were cultured to blastocysts for assessing their viability using Hoechst and PI staining. The viability rate was calculated based on the ratio of blue cells (as living cells) to the total cells. The rest of the two-cell embryos were collected for examining the expression of *Zp3*, *E-cad*, and *Ctnnb1* genes using molecular techniques.

### Gene expression

The synthetic cDNA was stored untill the polymerase chain reaction (PCR) stage at -80°C. Three genes (*Zp3, E-cad*, and *Ctnnb1*) were assessed by quantitative real-time PCR (qRT-PCR) in embryos from all groups. QuantiTect SYBER Green RT-PCR kit (Applied Biosystems, UK) using for calculation of gene expression. Primer sequence of experimental and reference genes (β-actin gene) are showed in Table I.

**Table 1:** Oligonucleotide primers in *ZP3*, *E-cadherin*, β-*catenin*, and β-*actin* genes


**Gene bank accession number**	**Gene**	**Sequence**	**Size (bp)**
**NM_011776.1**	*ZP3*	F: TCCCTTCAGAGCCACTGTGTCCTC R: GTGGAAGGTGGGAGCCGATTTC	100
**NM_009864.3**	*E-cadherin*	F: AGCCATTGCCAAGTACATCC R: AAAGACCGGCTGGGTAAACT	133
**NM_001165902.1**	β-*catenin*	F: ACCATCCACGCAGTTTGAC R: TCCTCATCGTTTAGCAGTTTTG	166
**NM_007393.5**	β-*actin*	F: GTACTCTGTGTGGATCGGTGG R: AACGCAGCTCAGTAACAGTCC	144

### Ethical considerations

This study was approved by the Ethics Board of Yazd Reproductive Sciences Institute‎, Iran (Code: IR.SSU.RSI.REC.1394.5). All animals were treated following the National Institutes of Health Guidance for the Care and Use of Laboratory Animals.

### Statistical analysis 

Data were analyzed by Graph pad prism 8.0.1 (GraphPad Software, Inc. San Diego, USA) and p < 0.05 was considered as significant. Moreover, the results of the oocyte and embryo parameters, and gene expression were calculated using two-way ANOVA. Tukey's post-test was used for the differences between the two groups. The differences between the quantitative parameters were compared using Chi-square test.

## 3. Results

**Figure 1:**
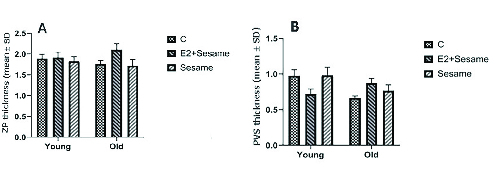
(A & B) Comparison of ZP and PVS thickness in control and treatment samples between old and young mice, respectively. P < 0.05 was considered as significant between the groups. Data were calculated using two-way ANOVA.

The mean thickness of the ZP and PVS was slightly higher in old mice that were treated with E2 + sesame oil compared to the control group (2.09 ± 0.46 vs. 1.75 ± 0.28 µm; 0.87 ± 0.22 µm vs. 0.65 ± 0.11 µm, respectively) (Figure 1). The mean number of oocytes was also increased in young and old groups that were treated with sesame oil. Data also showed that the number of two-cell embryos significantly increased in the old groups that were treated with sesame oil (Tables II). The number of blastocysts and viable cells was increased in the old group that was treated with sesame oil alone compared with the other old group. However, the young groups did not significantly differ in their embryo parameters (Tables II, III). In terms of the oocyte quantitative parameters, the normal ZP shape and the RF numbers were higher in the old group that was treated with seasame oil compared with the equivalent young group (Table IV).

The results showed that expression of the *Zp3* and *Ctnnb1* genes was not significantly different in the E2 and sesame samples compard with the controls, for the young or the old mice. However, the *E-cad* gene was downregulated in the sesame oil groups (p = 0.002 in young and p < 0.0001 in old mice) and in the E2 + sesame oil groups (p = 0.03 in young and p < 0.0001 in old mice) compared to the control groups.

**Table 2:** Comparison of embryo parameters between the three groups in young mice


**Young mice**	**Control**	**E2 + sesame oil**	**Sesame oil**	*p-value
**Oocyte (No.)**	25	24	35	0.06
**Two cells (%)**	59.16 ± 6.8	76.02 ± 4.4	68.7 ± 7.02	0.06${}^{a}$ 0.08${}^{b,}$ ${}^{c}$
**Blastocysts (%)**	39.3 ± 3.11	41.9 ± 3.9	43.2 ± 4.2	0.1${}^{a,}$ ${}^{b}$ 0.07${}^{c}$
**Viability rate (%)**	55.4 ± 5.8	65.2 ± 7.8	70.1 ± 6.2	0.07${}^{a}$ 0.08${}^{b}$ 0.05${}^{c}$
Data presented as percentage ± SD. *Two-way ANOVA. P-value < 0.05 was considered significant. E2: Estradiol. ${}^{a}$Difference between the control and E2 + sesame oil groups, ${}^{b}$Difference between the E2 + sesame oil and sesame oil alone groups, ${}^{c}$Difference between the control and sesame oil alone groups				

**Table 3:** Comparison of embryo parameters between the three groups in old mice


**Old mice**	**Control**	**E2 + sesame oil**	**Sesame oil**	*p-value
**Oocyte (No.)**	21	19	24	0.07
**Two cells (%)**	53.33 ± 7.2	62.7 ± 5.8	78.7 ± 6.4	0.06${}^{a,}$ ${}^{b}$ 0.018${}^{c}$
**Blastocysts (%)**	38.2 ± 4.1	42.5 ± 4.2	56.6 ± 5.2	0.06${}^{a}$ 0.04${}^{b}$ 0.002${}^{c}$
**Viability rate (%)**	56.5 ± 6.2	61.2 ± 6.8	92.08 ± 7.8	0.07${}^{a}$ 0 < 0001${}^{b,}$ ${}^{c}$
Data presented as percentage ± SD. *Two-way ANOVA. P-value < 0.05 was considered significant. E2: Estradiol. ${}^{a}$Difference between the control and E2 + sesame oil groups, ${}^{b}$Difference between the E2 + sesame oil and sesame oil alone groups, ${}^{c}$Difference between the control and sesame oil alone groups				

**Table 4:** The differential effect of sesame oil on ZP shape and refractile body in young and old mice


	**ZP shape**			**Refractile body**		
**Mice**	**Normal**	**Abnormal**	**p-value**	**Yes**	**No**	**p-value**
	**Young**	13 (43.3%)	7 (56.7%)	7 (23.3%)	23 (76.7%)	
**Old**	23 (76.7%)	7 (23.3%)	0.008*	16 (53.3%)	14 (46.7%)	0.016*
*P-value < 0.05 was considered as significant between young and old groups that were treated with sesame oil. ZP: Zona pellucida. The results are presented as percentage using Chi-square test						

## 4. Discussion

In the current study, the effects of E2 and sesame oil on oocyte morphological parameters and embryo formation were evaluated. Based on the results, the ZP thickness increased slightly in old mice that received E2 + sesame oil. Similar to our study, others showed that ZP size was thicker in zygotes when the E2 was high (1). This showed a possible relationship between the ZP thickness and the level of E2. They also reported that aged oocytes had thicker ZP with a reduced cytoplasm diameter (1). Due to inadequate nutrition of oocytes, both the quantity and maturation decreased in the oocytes. This process may happen in young mice; however, exogenous estrogen may be controlled by the activation of the gonadal, pituitary, hypothalamic axis which has less activity in the old group. Previous study showed that ZP thickness was significantly correlated with animal age. They concluded that a thicker ZP could be responsible for reducing the oocyte nutrition, fertilization, and embryo quality in older patients (20). Exogenous estrogen plays an important role in ZP morphology and oocyte nutrition (21). An increase in ZP thickness has a negative effect on the growth and maturation of oocytes when using E2 in old mice. Based on our data, the oocyte number decreased in old mice that received E2 + sesame oil. The main reason for the decrease in oocytes is assumed to be follicle regression after treatment with exogenous E2. Suppression of the dominant follicles' growth after E2 + sesame oil treatment has been documented before (22).

ZP structure abnormalities may directly reflect oocyte maturation defects (23). The follicular milieu determines the oocyte and early embryonic development (24). A balance and interaction between ROS production and antioxidant level are necessary for both folliculogenesis and oogenesis. In advanced age, antioxidant expression is diminished and, for this reason, the amount of ROS increase dramatically (25). Moreover, aging leads to a decrease in both E2 and antioxidant enzymes, such as catalase in follicular fluid. The failure in the balance produces free radicals which results in decreased oocyte quality and fertilization rate (26). Sesame oil is known as a natural antioxidant for oxidative stability (27). Our results showed that ZP normalcy increased in old mice that received sesame oil, while no improvement was documented when seasame oil was in combination with E2. The results also showed that the number of two-cell embryos, the blastocyst rate, and live cells increased in the old mice treated with sesame oil. A previous study showed that a low dose of E2 (1 µl/kg/day) had the best effect (15). The high dose of E2 (10 µl/kg/day) used in this study may have caused inconsistency in the homeostasis conditions. However, embryo parameters had better conditions in the sesame oil group than in the E2 + sesame oil group. In order to compensate for this defect, the use of antioxidants is recommended for older cases (28). In young infertile patients, the exogenous antioxidant may disrupt the balance with subsequent effects on oocyte growth and embryo development because of E2 production.

Some genes like *E-cad *play a critical role in oocyte maturation and embryonic development (29, 30). This connexin protein is associated with gap junctions between COCs and the oocyte. Folliculogenesis and fertilization depend on this gap junction (31). The expression of the *E-cad* gene was reduced in our young and old mice that had the E2 and sesame oil treatment. This is probably because these treatments have a similar function as *E-cad*, which disrupts the gene expression. Also, we report no significant difference in the expression of the *Zp3* and *ctnnb1* genes. This may be due to the estrogen or sesame oil properties, as greater effects were noted in oocyte morphology than in expressed genes. Jefferson and colleagues reported no effect of estrogen on gene expression in mice (32). In addition, in rats and mice, exogenic estrogen has been shown to postpone the development of the primordial follicles into primary follicles (33). In our study, sesame oil alone, compared to the E2 + sesame oil treatment, had a higher protective effect on oocyte morphological parameters, except for the RF. Contrary to our study, Otsuki and co-workers reported that RF was significantly associated with reduced fertilization rates. While the results showed that two-cell embryos, blastocysts, and viable cells increased in old mice treated with sesame oil, it is supposed that increasing RF has no effect on the fertilization rate and embryo development. Contrary to our study, Otsuki and colleagues reported that larger lipofuscin inclusions were associated with significantly reduced fertilization and unfavorable blastocyst development (34).

## 5. Conclusion

Using sesame oil along with exogenous E2 in old mice is not beneficial, because it increases the relative diameter of the ZP. However, the antioxidant properties of sesame oil can improve oogenesis, increasing oocyte quality and embryo development during aging. This may be beneficial in clinical settings in selecting compatible oocytes with subsequent elevation of IVF success rate.

##  Conflict of Interest

There is no conflict of interest.
